# Supplementary figures and images for: Spatiotemporal trends in bed bug metrics: New York City
Source: PLoS One. 2022 May 26;17(5):e0268798. doi: 10.1371/journal.pone.0268798 (PMC9135212; doi:10.1371/journal.pone.0268798)

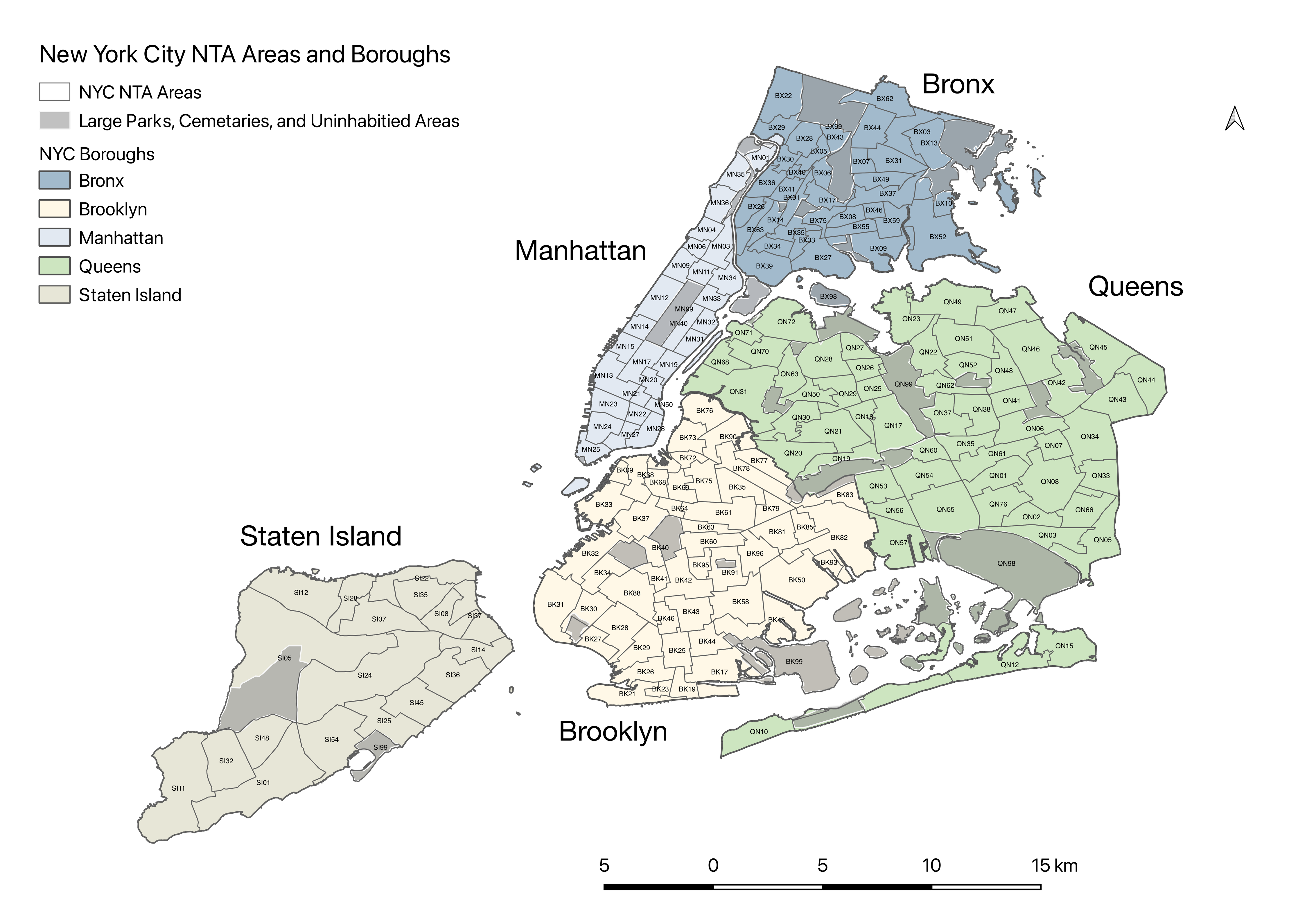

Supplement: S1 Fig — A map displaying the NTA areas of New York City within their respective boroughs. NTA areas are designated with their NTA code, the respective neighborhoods associated with each NTA code are available in S1 File. (TIF) [file pone.0268798.s001.tif]

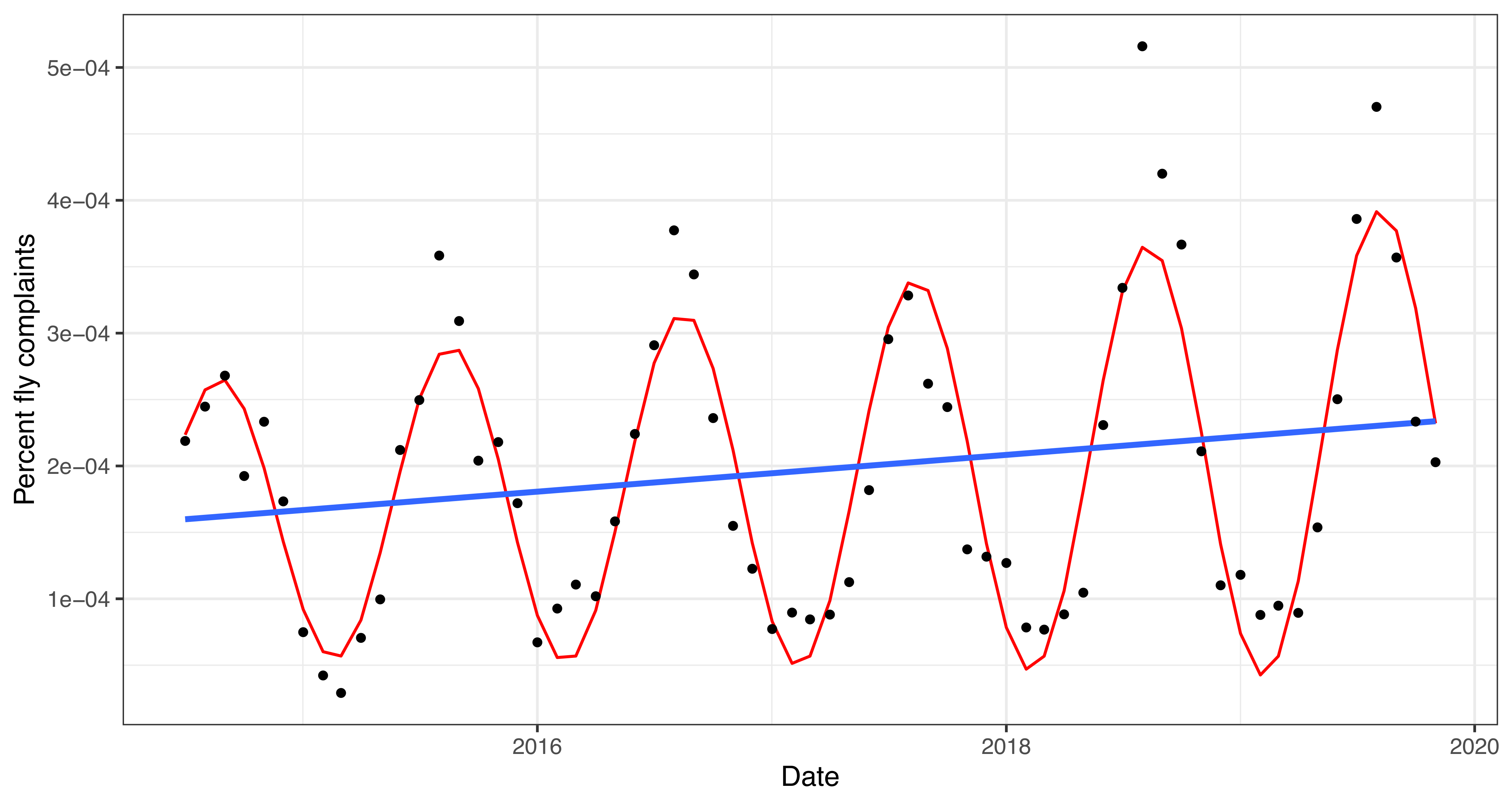

Supplement: S2 Fig — Graphical representation of the results of a linear harmonic model assessing the temporal relationship of official fly complaints from 2014–2019. Fly complaints modeled as a linear harmonic model with decreasing amplitude over time. (TIF) [file pone.0268798.s002.tif]

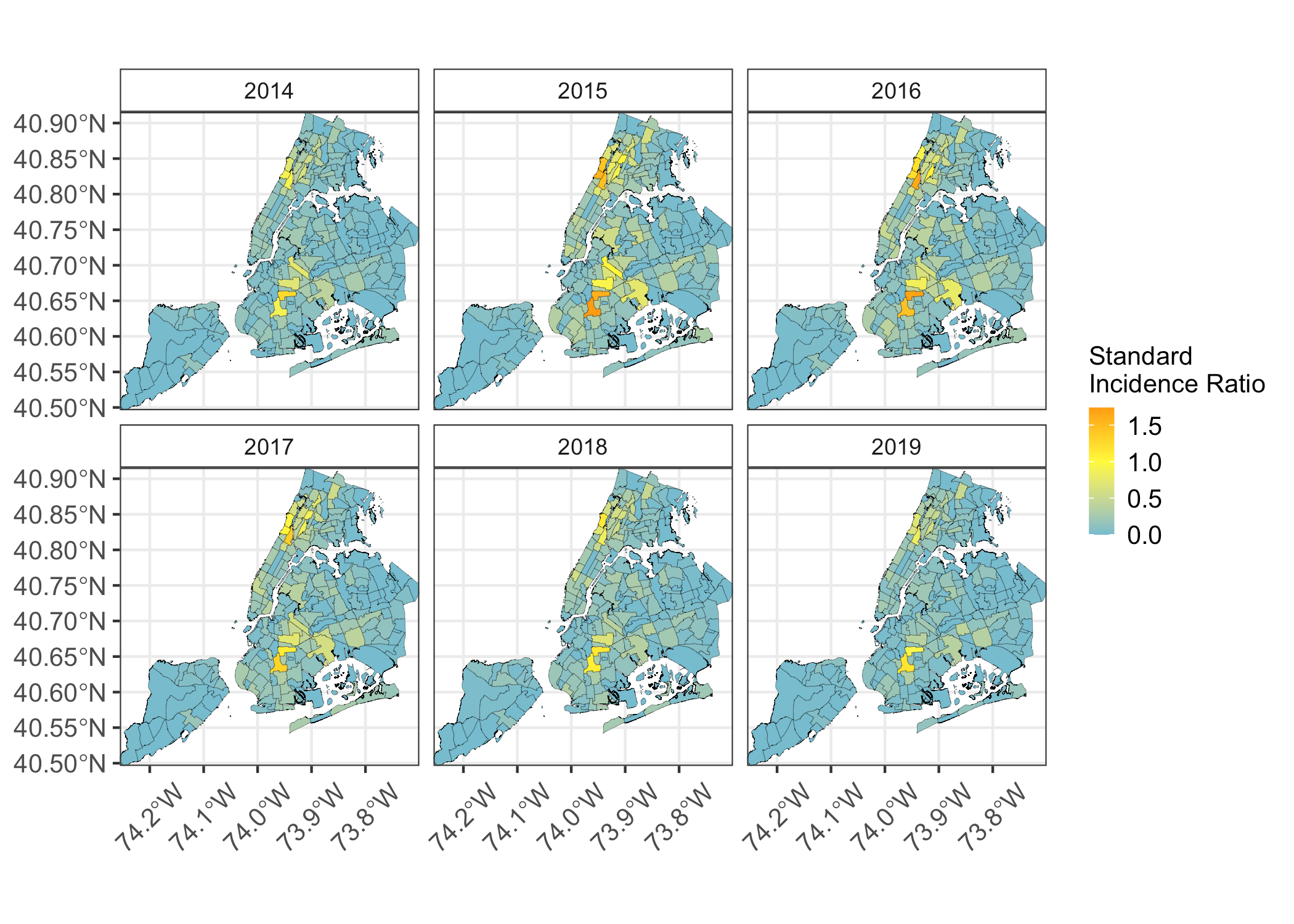

Supplement: S3 Fig — Standard Incident Ratio was calculated as the ratio between the observed and expected number of bed bug complaints per NTA area. Expected accounts were calculated via indirect standardization. Specific NTA area names per borough are listed in S1 File. (TIF) [file pone.0268798.s003.tif]

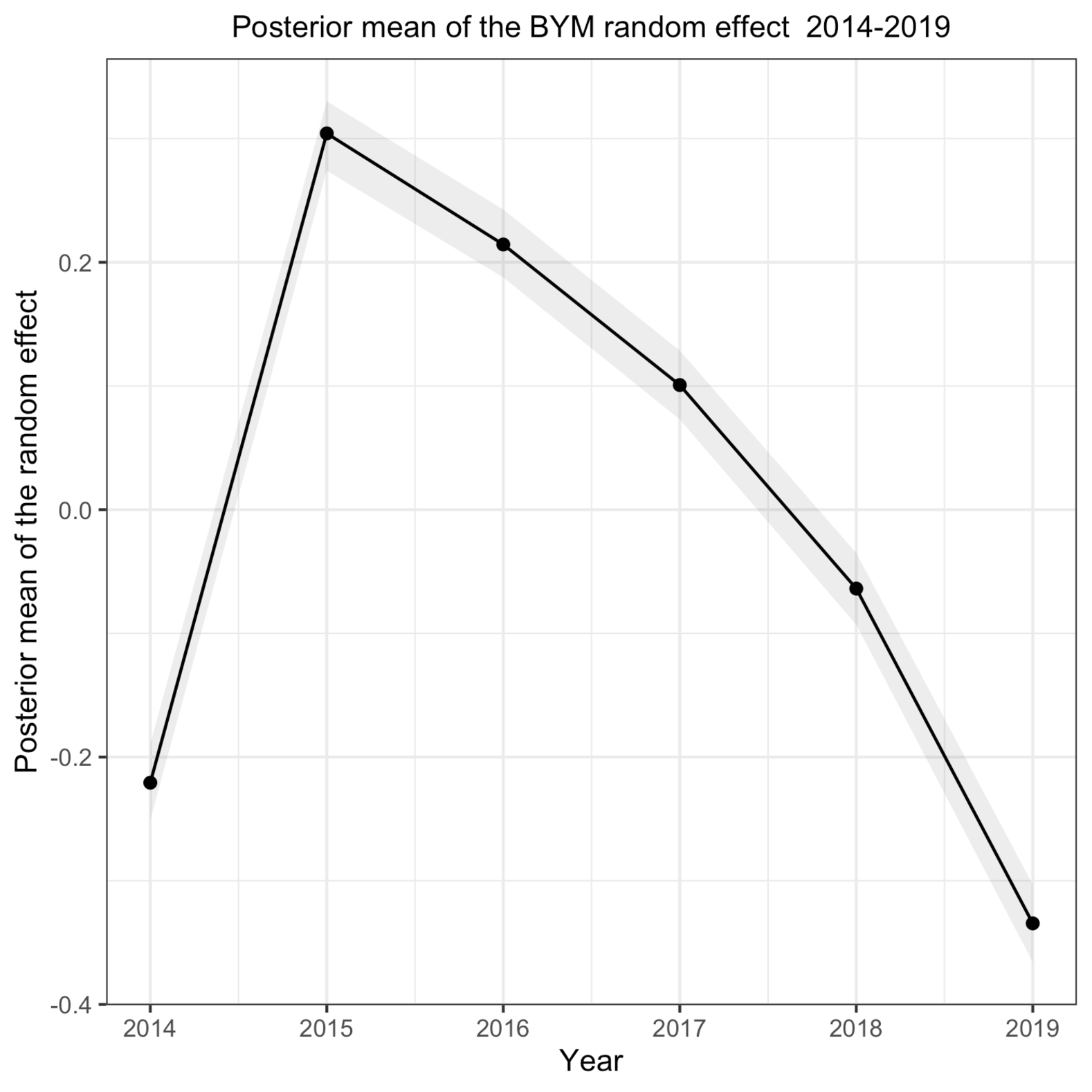

Supplement: S4 Fig — (TIF) [file pone.0268798.s004.tif]

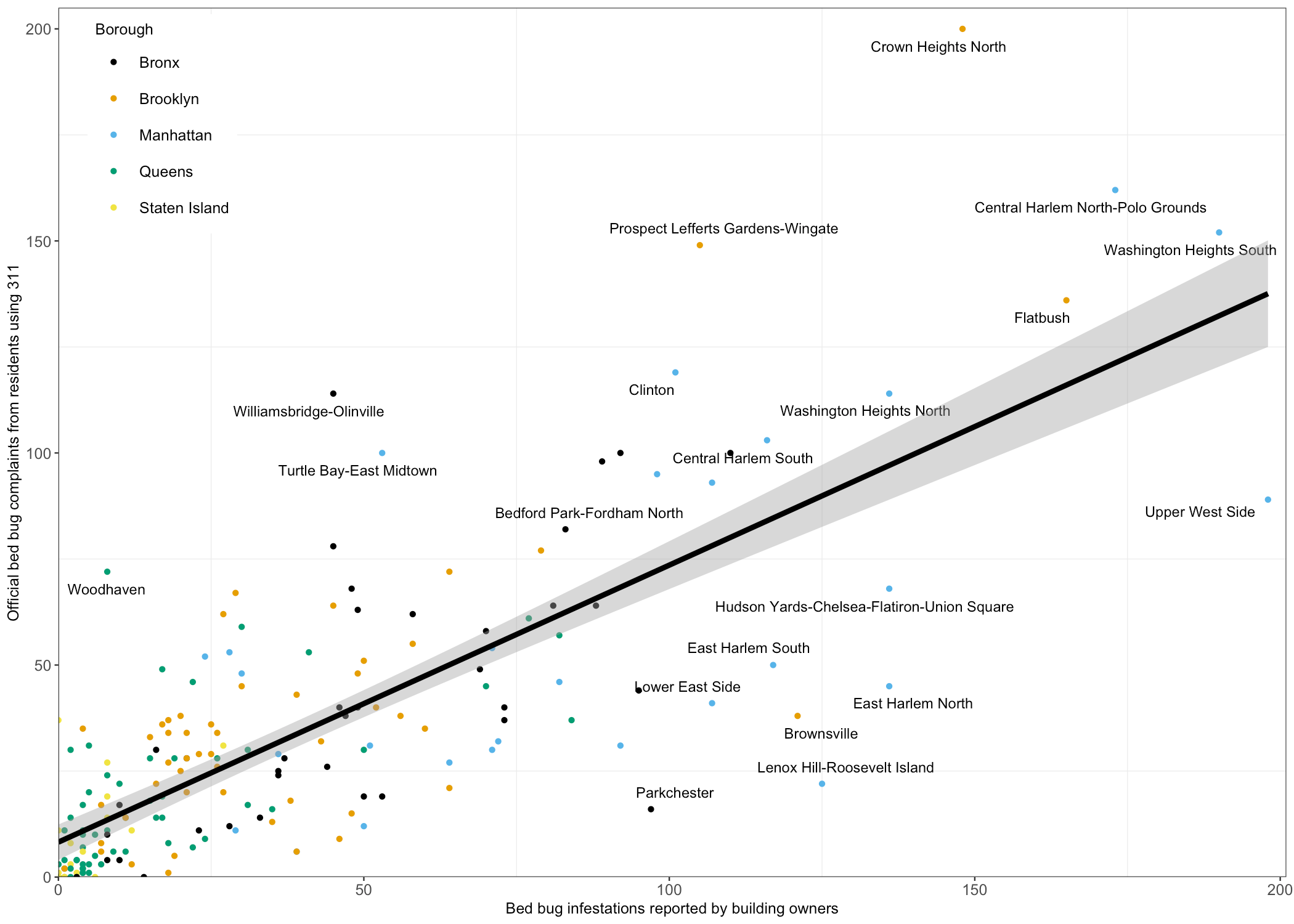

Supplement: S5 Fig — Correlation between official bed bug complaints from residents (n = 6376) and building manager reported infestation (n = 7303) was high (R2 = 0.60). (TIF) [file pone.0268798.s005.tif]
