# Supplementary material for: Spatiotemporal trends in bed bug metrics: New York City
Source: PLoS One. 2022 May 26;17(5):e0268798. doi: 10.1371/journal.pone.0268798 (PMC9135212; doi:10.1371/journal.pone.0268798)
Supplement: S1 Table — Basic demographic statistics for the five boroughs of New York City. Information for table was obtained through the U.S. Census Bureau QuickFacts resource [49]. (DOCX) [file pone.0268798.s006.docx]

**Supplemental Table 1.** **New York City Boroughs and Basic Statistics**.

| Borough | Number of NTA areas | Population Census | | Population per Square Mile | Median Household Income | Percent Living in Poverty |
| --- | --- | --- | --- | --- | --- | --- |
|  |  | 2010 | 2020 |  |  |  |
| Manhattan | 29 | 1,694,251 | 1,585,873 | 69,467.5 | $86,553 | 16.3% |
| Queens | 58 | 2,230,722 | 2,405,464 | 20,553.6 | $68,666 | 10.3% |
| Brooklyn | 51 | 2,504,700 | 2,736,074 | 35,369.1 | $60,231 | 17.8% |
| Bronx | 38 | 1,385,108 | 1,472,654 | 32,903.6 | $40,088 | 24.7% |
| Staten Island | 20 | 468,730 | 495,747 | 8,030.3 | $82,783 | 10.6% |

Basic demographic statistics for the five boroughs of New York City. Information for table was obtained through the U.S. Census Bureau QuickFacts resource.

U.S. Census Bureau QuickFacts: Queens County, New York; New York County, New York; United States. In: U.S. Census Bureau QuickFacts [Internet]. [cited 17 Jan 2022]. Available: https://www.census.gov/quickfacts/fact/table/richmondcountynewyork,bronxcountynewyork,kingscountynewyork,queenscountynewyork,newyorkcountynewyork,US/PST045221
