# Supplementary material for: Spatiotemporal trends in bed bug metrics: New York City
Source: PLoS One. 2022 May 26;17(5):e0268798. doi: 10.1371/journal.pone.0268798 (PMC9135212; doi:10.1371/journal.pone.0268798)
Supplement: S2 Table — Database descriptions including timeframe, georeferencing information, organizational management of database, and codes used for analysis. (DOCX) [file pone.0268798.s007.docx]

| Database | Timeframe Available | Georeferencing Information | Description | Code or text search used | Department |
| --- | --- | --- | --- | --- | --- |
| 311 Inquires | 2010–2019 | Not georeferenced | All calls, online inquiries, and inquiries registered through the app | N/A | NYC 311 |
| 311 bed bug specific requests^a^ | 2010–2019 | Not georeferenced | Official bed bug complaints, as well as general bed bug inquires | Text search using bed bug terms^b^ | NYC 311 |
| Official bed bug complaints^c^ | 2014–2019 | Georeferenced | Official bed bug complaints registered with  HPD^d^ | Problem Category Values:  2818 & 2517 | HPD |
| Official cockroach complaints | 2014–2019 | Georeferenced | Official cockroach complaints registered with  HPD | Problem Category Values: 2514  & 2823 | HPD |
| Building owner reported bed bug infestations^e^ | 2018 | Georeferenced | Units infested by bed bugs as reported by building managers to the HDP | Infested units | HPD |

**Supplemental Table 2.** **Descriptive properties of each of the datasets**.

Database descriptions including timeframe, georeferencing information, organizational management of database, and codes used for analysis

^a^311 bed bug specific requests are a subset of general 311 inquiries extracted through text search

^b^ Text search terms included: bedbug(s), bed bug(s), and pest(s). Pests according to the housing maintenance code include bed bugs as well as “any unwanted member of class Insecta.”

^c^ Formal bed bug complaints have been archived by the HDP since 2014 and are a subset of 311 inquiries

^d^ Department of Housing and Preservation

^e^ Report obtained by a freedom of information request
