# Supplementary material for: Spatiotemporal trends in bed bug metrics: New York City
Source: PLoS One. 2022 May 26;17(5):e0268798. doi: 10.1371/journal.pone.0268798 (PMC9135212; doi:10.1371/journal.pone.0268798)
Supplement: S3 Table — Description of bed bug related inquires and the agencies that processed the request. (DOCX) [file pone.0268798.s008.docx]

Supplemental Table 3. Total number of inquiries processed by New York City’s 311 that included bed bugs as part of the description from 2010-2019.

| Bed Bug Related Inquires | Departments Handling 311 Inquiries Regarding Bed Bugs (n = 246629) | | | | | | | |
| --- | --- | --- | --- | --- | --- | --- | --- | --- |
| Inquiry Description | 3-1-1 Call Center | CUNY^d^ | DOE^e^ | DOHMH^f^ | HPD^g^ | HRA^h^ | MTA^i^ | NYCHA^j^ |
| BB^a^ at CUNY^d^ College | 0 | 39 | 0 | 0 | 0 | 0 | 0 | 0 |
| Pests^b^ in Apartment | 0 | 0 | 0 | 0 | 29479 | 0 | 0 | 0 |
| Pests^b^ in Residential Public Area | 0 | 0 | 0 | 0 | 6479 | 0 | 0 | 0 |
| BB^a^ complaint from a tenant in a residential building | 0 | 0 | 0 | 0 | 46886 | 0 | 0 | 0 |
| BB^a^ complaint in business or nonprofit | 4423 | 0 | 0 | 0 | 0 | 0 | 0 | 0 |
| BB^a^ complaint in domestic violence shelter | 0 | 0 | 0 | 0 | 0 | 270 | 0 | 0 |
| BB^a^ complaint in hotel or SRO^c^ | 0 | 0 | 0 | 0 | 1125 | 0 | 0 | 0 |
| BB^a^ complaint in NYC school | 1 | 0 | 4596 | 0 | 0 | 0 | 0 | 0 |
| BB^a^ complaint in NYC housing authority. | 2 | 0 | 0 | 0 | 0 | 0 | 0 | 12589 |
| Complaint that might attract pests^b^ in subways or other MTA authority | 0 | 0 | 0 | 0 | 0 | 0 | 484 | 0 |
| BB^a^ complaint in daycare centers | 12 | 0 | 0 | 418 | 0 | 0 | 0 | 0 |
| Get information about BB^a^ in day care centers | 0 | 0 | 0 | 49 | 0 | 0 | 0 | 0 |
| Get information about how to comply with health commissioner’s order about BB^a^ | 3 | 0 | 0 | 2072 | 0 | 0 | 0 | 0 |
| Information for preventing and getting rid of BB^a^ | 0 | 0 | 0 | 480 | 0 | 0 | 0 | 0 |
| Make a complaint about BB^a^ in a residential building hotel or SRO^c^ building | 0 | 0 | 0 | 0 | 106096 | 0 | 0 | 0 |
| Request a copy of BB^a^ prevention and control brochure | 49 | 0 | 0 | 30939 | 0 | 0 | 0 | 0 |
| Information about filing BB annual report for building owners online | 0 | 0 | 0 | 0 | 138 | 0 | 0 | 0 |
| Total | 4484 | 39 | 4596 | 33958 | 190203 | 270 | 484 | 12589 |

Description of bed bug related inquires and the agencies that processed the request.

^a^Bed Bugs

^b^Bed bugs, fleas, flies, roaches, mice, or other pests

^c^Single Room Occupancy

^d^City University of New York

^e^Department of Education

^f^Department of Health and Mental Hygiene,

^g^Department of Housing Preservation and Development

^h^Human Resources Administration

^i^Metropolitan Transit Authority

^j^New York City Housing Authority
