# Supplementary material for: Spatiotemporal trends in bed bug metrics: New York City
Source: PLoS One. 2022 May 26;17(5):e0268798. doi: 10.1371/journal.pone.0268798 (PMC9135212; doi:10.1371/journal.pone.0268798)
Supplement: S4 Table — Model results of a linear harmonic model assessing the association between month and number of official bed bug complaints and cockroach complaints from 2014–2019. Bed bug and cockroach complaints were standardized by the total number of 311 inquiries to obtain percentages. (DOCX) [file pone.0268798.s009.docx]

**Supplemental Table 4. Association between time and official bed bug and cockroach complaints accounting for seasonality.**

| Variable | Estimate | Confidence Interval | p-value |
| --- | --- | --- | --- |
| Bed Bug Complaints |  |  |  |
| Time^a^ | -1.8 e -5 | -2.4e-5– -1.2e-5 | p < .01 |
| Amplitude^b^ | 0.3 | 0.1–0.5 | p < .01 |
| Phase Shift^a^ | 1.4 | 0.8–2.0 | p < .01 |
| Cockroach Complaints |  |  |  |
| Time^a^ | 7.8e-5 | 6.5e-5–8.9e-5 | p < .01 |
| Amplitude^b^ | 0.3 | 0.1­–0.4 | p < .01 |
| Phase Shift^a^ | 1.4 | 0.8–2.0 | p < .01 |

Model results of a linear harmonic model assessing the association between month and number of official bed bug complaints and cockroach complaints from 2014 – 2019. Bed bug and cockroach complaints were standardized by the total number of 311 inquiries to obtain percentages.

^a^Measured in months

^b^Measured in the number of complaints per total number of 311 inquires multiplied by 100,000
