# Supplementary material for: Spatiotemporal trends in bed bug metrics: New York City
Source: PLoS One. 2022 May 26;17(5):e0268798. doi: 10.1371/journal.pone.0268798 (PMC9135212; doi:10.1371/journal.pone.0268798)
Supplement: S5 Table — (DOCX) [file pone.0268798.s010.docx]

Supplemental Table 5. Bed bug complaints throughout the boroughs as processed by HPD by year.

| Borough | Year | Number of Bed Bug Complaints | Estimated Population | Proportion per 100,000 ± (95% Confidence Interval) |
| --- | --- | --- | --- | --- |
| Bronx | Total | 10,394 | 1,434,093 | 724.8 ± CI (711, 738.8) |
|  | 2014 | 1,330 | 1,413,566 | 94.09 ± CI (89.17, 99.28) |
|  | 2015 | 2,337 | 1,428,357 | 163.6 ± CI (157.1, 170.4) |
|  | 2016 | 1,993 | 1,436,785 | 138.7 ± CI (132.8, 144.9) |
|  | 2017 | 1,968 | 1,455,846 | 135.2 ± CI (129.3, 141.3) |
|  | 2018 | 1,561 | 1,437,872 | 108.6 ± CI (103.3, 114.1) |
|  | 2019 | 1,205 | 1,432,132 | 84.14 ± CI (79.52, 89.03) |
| Brooklyn | Total | 14,544 | 2,598,602 | 559.7 ± CI (550.7, 568.8) |
|  | 2014 | 2,009 | 2,570,801 | 78.15 ± CI (74.8, 81.64) |
|  | 2015 | 3,300 | 2,595,259 | 127.2 ± CI (122.9, 131.6) |
|  | 2016 | 2,925 | 2,606,852 | 112.2 ± CI (108.2, 116.3) |
|  | 2017 | 2,589 | 2,635,121 | 98.25 ± CI (94.54, 102.1) |
|  | 2018 | 2,172 | 2,600,747 | 83.51 ± CI (80.08, 87.1) |
|  | 2019 | 1,549 | 2,582,830 | 59.97 ± CI (57.06, 63.03) |
| Manhattan | Total | 9,399 | 1,632,991 | 575.6 ± CI (564.1, 587.3) |
|  | 2014 | 1,134 | 1,618,389 | 70.07 ± CI (66.11, 74.27) |
|  | 2015 | 2,069 | 1,629,507 | 127 ± CI (121.6, 132.6) |
|  | 2016 | 1,989 | 1,634,989 | 121.7 ± CI (116.4, 127.1) |
|  | 2017 | 1,683 | 1,653,877 | 101.8 ± CI (97.02, 106.7) |
|  | 2018 | 1,454 | 1,632,480 | 89.07 ± CI (84.61, 93.76) |
|  | 2019 | 1,070 | 1,628,701 | 65.7 ± CI (61.88, 69.75) |
| Queens | Total | 6,485 | 2,301,409 | 281.8 ± CI (275, 288.7) |
|  | 2014 | 812 | 2,280,602 | 35.6 ± CI (33.24, 38.14) |
|  | 2015 | 1,374 | 2,301,139 | 59.71 ± CI (56.64, 62.95) |
|  | 2016 | 1,303 | 2,310,011 | 56.41 ± CI (53.43, 59.55) |
|  | 2017 | 1,118 | 2,339,280 | 47.79 ± CI (45.07, 50.68) |
|  | 2018 | 1,032 | 2,298,513 | 44.9 ± CI (42.24, 47.72) |
|  | 2019 | 846 | 2,278,906 | 37.12 ± CI (34.7, 39.71) |
| Staten Island | Total | 907 | 473,926 | 191.4 ± CI (179.3, 204.2) |
|  | 2014 | 145 | 471,522 | 30.75 ± CI (26.13, 36.19) |
|  | 2015 | 166 | 472,481 | 35.13 ± CI (30.18, 40.9) |
|  | 2016 | 187 | 473,324 | 39.51 ± CI (34.23, 45.59) |
|  | 2017 | 157 | 475,948 | 32.99 ± CI (28.21, 38.57) |
|  | 2018 | 163 | 474,101 | 34.38 ± CI (29.49, 40.08) |
|  | 2019 | 89 | 476,179 | 18.69 ± CI (15.18, 23.01) |
