# Supplementary material for: Spatiotemporal trends in bed bug metrics: New York City
Source: PLoS One. 2022 May 26;17(5):e0268798. doi: 10.1371/journal.pone.0268798 (PMC9135212; doi:10.1371/journal.pone.0268798)
Supplement: Attachment — Submitted filename: Responces to Reviewers.docx [file pone.0268798.s012.docx]

**Academic Editor:**

Correct the references according to the recommended format of the Journal

We have corrected the references according to the style guide of PLOS One.

Add latest references as pointed out in the reviewer’s comments

We have updated the reference section to include additional references as suggested by Reviewer 1.

Check the supplementary data according to the reviewer's comments

We have updated the supplementary data according to the reviewer’s comments.

Improve the conclusive remarks

We have updated conclusive remarks and performed an additional control analysis according to recommendations.

**Reviewer #1:**

The New York City area has thousands of licensed pest management professionals and hundreds of pest control companies. Could these results have been explained by a mere shift from the public to the private sector?

The reviewer raises an important question. New York City does not handle bed bug treatment, only inspection. Private pest control has always handled bed bug treatment, apart from New York public housing which was not included in this analysis.

 However, it is possible that New York City tenants are reporting fewer bed bug complaints due to landlords being more responsive to building infestation as a result of the new laws in New York City. While it is possible that tenants may choose not to report, for 2018 we have reporting from building managers and there is good concordance between building manager reports and official New York City complaints (S5 Fig. 5).

We have added additional text to the discussion to address this concern (463-466). Text added below for ease:

(463-466): Our study was not without limitations. Self-reported and landlord reporting of bed bug infestation have inherent biases and inaccuracies [42]. It is possible that tenants have started to report fewer complaints through the city and instead communicate directly with their building manager. However, for the records we have available we see high concordance between infestations reported by building managers and those reported to the city by tenants.

Reference 3 looks incomplete (year of publication).

Corrected

References 1-4: more recent references could be added.

Additional references were added specifically:

Akhoundi M, Sereno D, Durand R, Mirzaei A, Bruel C, Delaunay P, et al. Bed Bugs (Hemiptera, Cimicidae): Overview of Classification, Evolution and Dispersion. International Journal of Environmental Research and Public Health. 2020;17: 4576. doi:10.3390/ijerph17124576

Crawley SE, Borden JH. Detection and monitoring of bed bugs (Hemiptera: Cimicidae): review of the underlying science, existing products and future prospects. Pest Management Science. 2021;77: 5334–5346. doi:10.1002/ps.6574

Lowe EC, Latty T, Webb CE, Whitehouse MEA, Saunders ME. Engaging urban stakeholders in the sustainable management of arthropod pests. J Pest Sci. 2019;92: 987–1002. doi:10.1007/s10340-019-01087-8

Raab RW, Moore JE, Vargo EL, Rose L, Raab J, Culbreth M, et al. New Introductions, Spread of Existing Matrilines, and High Rates of Pyrethroid Resistance Result in Chronic Infestations of Bed Bugs (Cimex lectularius L.) in Lower-Income Housing. Benoit JB, editor. PLoS ONE. 2016;11: e0117805. doi:10.1371/journal.pone.0117805

Could one single bedbug complaint be registered in more than one database?

Bed bug complaints can be registered in both the general 311 New York City database as well as the official bed bug complaint database. However, the data were never pooled in analysis. We added additional text to clarify this (147-149).

Text added below for ease:

(147-149): The specifics of each database (attributes, time scales, and geographic information) are summarized in S2 Table 2 and described in detail below. All databases were analyzed individually, and no data sources were pooled during analysis.

Why was the year 2020 not included? It could have been interesting to study the effect of confinement.

Unfortunately, during the covid crisis many of New York City’s open data information portals were not updated regularly due to shutdowns and quarantines of government staff and during the time of this analysis the data from 2020 was unavailable.

**Reviewer #2:**

In suppl. material file, Suppl. Table 5 is repeated with titles "Modeling framework used to assess potential space-time interactions of bed bug complaints per NTA area" and "Results of the nonparametric spatiotemporal model (model 1) without space-time interaction effects". These might be Tables 5 and 6. I suggest that Tables 5 and 6 may be included in the results of main MS along with Suppl. Figs 1 and 3. However, it depends if the authors agree.

We agree with the reviewer and added Table 5 and 6 and Figs 1 and 3 to the main text.

**Reviewer #3:**

 My only concern is about the main conclusion: the number of bed bugs complains decreased, while cockroach complaints increased from 2014 based on data period of 2010-2019. The decrease in bed bug complaints is not surprising. Increased awareness, better methods and materials and control strategies, regulations, fatigue in reporting bed bugs might have contributed to the decline of bed bug complaints. The conclusion about increasing cockroach complaints is surprising. There is no evidence from pest control industry that shows cockroach infestations are increasing in the last 5-7 years. There are no revolutionary novel products appeared for cockroach control since 2014.

I do not see a reason why cockroach infestations increased after 2014. Authors need to re-investigate the data and interpret the results more cautiously.

The reviewer raises an important concern. We used cockroach complaints primarily as a control for reporting trends in insects. Our goal was to show that the decline in bed bug reports was not explained by an overall decline in reporting for insects, since reports for other insects such as cockroaches did not decrease in the same period. We agree that the increase in cockroach complaints was surprising. We ran an additional analysis for fly complaints for the same period. We observed the same seasonal reporting as we did for bed bugs and cockroaches (peaking in the summer and decreasing during winter), however average reporting for flies has not increased or decreased over the period, again suggesting that a decrease in overall insect reporting was not the cause of the decline in bed bugs.

We additionally updated the results and discussion to include information for fly complaints (73- 78), (175 - 181) and (378-391). Text and additional figure added below for ease:

(73- 78): Bed bug complaints have significantly decreased (p < 0.01) from 2014­–2020, the absolute number of complaints per month dropping by half (875 average complaints per month to 440 average complaints per month); conversely, complaints for other insects including cockroaches and flies did not decrease over the same period.

(175 - 181): Cockroaches and flies, like bed bugs, are persistent urban pests whose control relies on communication between tenant and landlord. Importantly, cockroach and fly infestation reporting are identical to bed bug reporting. However, unlike bed bugs there is no new legislation or policies that specifically target cockroach or fly infestation. Based on these characteristics, we use cockroach and fly complaints as a control-type groups which will enable us to identify potential temporal changes in insect reporting, in the absence of a true control area or population.

(378-391): During the time periods assessed, both general 311 inquiries about bed bugs (2010–2019) and official bed bug complaints (2014–2019) decreased in New York city. This decrease was significant (p < 0.001) both when standardized by population and against the totality of 311 inquiries and their decline in reporting was not explained by insect reporting generally. When compared to patterns observed in cockroach and fly complaints, which stand as imperfect insect control populations, the decrease in bed bug complaints is notable. The contrasting pattern of bed bug complaints, as well as strong seasonal patterns, indicate that the decrease in complaints is not an artifact of overall 311 or reporting use as these trends continued to be significant following standardization. While surprisingly little is known regarding the seasonality of bed bugs[38], the seasonal trends observed where bed bug complaints peaked in the summer and decreased during the winter is consistent with other studies of bed bug seasonality[39–41] and other reporting trends noted in the United States[38,42]. Ultimately, disclosure laws, new approaches in pest management[27], increased knowledge, and commitment to inspections have likely all contributed to this decrease in bed bug complaints in New York City.

S2 Fig. 2. Longitudinal analysis of official fly registered to HPD**.** Graphical representation of the results of a linear harmonic model assessing the temporal relationship of official fly complaints from 2014–2019. Fly complaints modeled as a linear harmonic model with decreasing amplitude over time.

Line 90-96. Need to describe the 5 boroughs being studied.

We added additional information in line 138-142 on the New York City Boroughs. We also included a supplemental table (S1 Table 1) and figure (S1 Fig 1) along with a supplemental file (S1 File 1) that summarizes and maps the areas used in this study.

(138-142): The area referred to as New York City is administratively organized into five “boroughs”: Manhattan, Brooklyn, Queens, Bronx, and Staten Island (S1 Fig 1), which are each designated as their own counties by the State of New York. These counties are a subgovernment units designated by the State of New York. We report general characteristics and demographics for these boroughs in S1 Table 1.

S1. Table 1. New York City Boroughs and Basic Statistics. Basic demographic statistics for the five boroughs of New York City. Information for table was obtained through the U.S. Census Bureau QuickFacts resource [44].

| Borough | Number of NTA areas | Population Census | | Population per Square Mile | Median Household Income | Percent Living in Poverty |
| --- | --- | --- | --- | --- | --- | --- |
|  |  | 2010 | 2020 |  |  |  |
| Manhattan | 29 | 1,694,251 | 1,585,873 | 69,467.5 | $86,553 | 16.3% |
| Queens | 58 | 2,230,722 | 2,405,464 | 20,553.6 | $68,666 | 10.3% |
| Brooklyn | 51 | 2,504,700 | 2,736,074 | 35,369.1 | $60,231 | 17.8% |
| Bronx | 38 | 1,385,108 | 1,472,654 | 32,903.6 | $40,088 | 24.7% |
| Staten Island | 20 | 468,730 | 495,747 | 8,030.3 | $82,783 | 10.6% |

Line 165. What does the relative risk scale mean? Need to explain.

Relative Risk is a scale where one is the average number of bed bug complaints predicted for the NTA. Values over one indicate a higher than average number of bed bug complaints and values under one indicate a lower than average number of bed bug complaints. Specifically, relative risk is defined as the posterior mean of the spatial temporal interaction *d_it_* for bed bug complaints per NTA area. We added additional text to indicate this (230-231). We additionally added to the text of the figure legend.

Line 192. Supplemental table 4 does not show seasonal data.

Due to space constraints we only described the total number of complaints by borough per year. Seasonality of complaints by borough is demonstrated by Figure 3.

Line 200. Supplemental Fig. 1 does not show the names of these boroughs.

We edited the caption of Fig. 1 to reflect that this captures all New York City boroughs.

Line 227. "R2" The number 2 should be in upper case

Corrected

Line 245-246. Delete the discussion about canine. It is not an effective bed bug detection method.

Removed

References. Need to check the styles.

Corrected

Fig. 4 legend. Need to be more descriptive. Please indicate this is for NYC. Explain the scales. This figure has lower resolution than the supplementary Figure.

We added additional text to the Figure 4 Legend to indicate that this is for New York City.

We added additional text to the methods section (126 -131) to indicate that the scale shows the relative risk of bed bug infestation based on the spatiotemporal model where relative risk is the posterior mean of the spatial temporal interaction *d_it_* for bed bug complaints per NTA area assuming no spatial and/or temporal structure.

(126-131): We then used a Bayesian Hierarchal modeling approach using Integrated Nested Laplace Approximation (INLA), that assessed the relative risk of bed bug complaints per NTA area, where the risk of the bed bug complaints in a given NTA area is compared to the expected number of complaints per NTA area given the population [36]. Relative risk is defined by the spatiotemporal model as the posterior mean of the spatial temporal interaction *δ_it_* for bed bug complaints per NTA area.

We fixed the resolution issue.

Supplemental Table 2. Need to add more details so readers know this is about NYC and only during 2020-2019. This Table shows 46886 bed bug complaints from residents to HPD during 2020-2019. If divided by 10, the number is still very large. Why supplemental figure shows very low number of complaints in 2018?

We have updated the title of Supplemental Table 2 to indicate it is all New York City 311 calls from 2010-2019.

Many of the dots in supplemental figure three were overlain, creating the impression that there were fewer reports. There were over 6376 complaints registered in 2018.

We have altered the symbology to make it clear that there are many points in the lower section of the y-axis and added the total number of complaints as part of the figure description.

**S5 Fig. 5. Comparison between building manager reported bed bug infestation and official bed bug complaints registered to HPD for 2018.** Correlation between official bed bug complaints from residents (n = 6376) and building manager reported infestation (n = 7303) was high (R^2^ = 0.60).

Supplemental Table 3. “Cockroach complaints” need to be moved further to the left.

Corrected

Supplemental Fig. 1. Use smaller font for x-axis legend. The “standard incidence ratio” legend needs to match with that for Fig. 4.

We changed the x-axis to be more legible. We also updated the colors on these graphics to help assess the gradations in the color values.

Supplemental Fig. 1 depicts the raw data expressed as the standard incidence ratio which is the ratio between the observed and expected counts of bed bug complaints per NTA area. Fig 4 shows the results of the spatiotemporal model and visually shows the posterior mean of the spatiotemporal model expressed as relative risk.

**S3 Fig. 3. Standard Incident Ratio (SIR) of official bed bug complaints per NTA area for all New York City Boroughs from 2014­­–2019.** Standard Incident Ratio was calculated as the ratio between the observed and expected number of bed bug complaints per NTA area. Expected accounts were calculated via indirect standardization. Specific NTA area names per borough are listed in S1 File 1.
