# Supplementary material for: Spatiotemporal trends in bed bug metrics: New York City
Source: PLoS One. 2022 May 26;17(5):e0268798. doi: 10.1371/journal.pone.0268798 (PMC9135212; doi:10.1371/journal.pone.0268798)
Supplement: Attachment — Submitted filename: Response to Reviewers.docx [file pone.0268798.s013.docx]

**Academic Editor:**

Journal Requirements:

Please review your reference list to ensure that it is complete and correct.

We have assessed the reference list and corrected the numbering which contained non-sequential numbering, and corrected typographical errors found in the references.

Additionally, we added two references which were more updated than the original listed references. The articles added are:

Bedbugs: Information for Tenants and Building Owners - NYC Health. [cited 25 Apr 2022]. Available: <https://www1.nyc.gov/site/doh/health/health-topics/bedbugs-information-for-landlords-and-building-managers.page>

Doggett SL, Geary MJ, Russell RC. The Resurgence of Bed Bugs in Australia: with Notes on Their Ecology and Control. Environmental Health 2004;4: 30-38.

If you have cited papers that have been retracted, please include the rationale for doing so in the manuscript text or remove these references and replace them with relevant current references. Any changes to the reference list should be mentioned in the rebuttal letter that accompanies your revised manuscript. If you need to cite a retracted article, indicate the article’s retracted status in the References list and also include a citation and full reference for the retraction notice.

While we did not note any journal retractions in our citation list, the article below did have a posted correction which updated their funding information. We added a “corrected version” to our citation list for clarity.

Leulmi H, Bitam I, Berenger JM, Lepidi H, Rolain JM, Almeras L, et al. Competence of Cimex lectularius Bed Bugs for the Transmission of Bartonella quintana, the Agent of Trench Fever. Corrected Version. PLoS Negl Trop Dis. 2015;9: e0003789. doi:10.1371/journal.pntd.0003789

**Reviewer Comments:**

We corrected all the following minor revisions noted by the authors.

Line 273. Add a space before =.

Corrected

Reference section: The scientific names (Cimex lectularius) need to be in italic font.

Corrected

Line 426-428. Two references with same number. One of them should be 7

Corrected, and fixed additional numbering errors in the citation list.

Line 525. Missing page number.

Corrected

Line 538. May need to delete "Jul-Aug". Also delete "(1)".

Corrected
